# Penetration mechanics of elongated female and male genitalia of earwigs

**DOI:** 10.1038/s41598-021-86864-1

**Published:** 2021-04-12

**Authors:** Yoko Matsumura, Yoshitaka Kamimura, Chow-Yang Lee, Stanislav N. Gorb, Hamed Rajabi

**Affiliations:** 1 Department of Functional Morphology and Biomechanics, Zoological Institute, Kiel University, Am Botanischen Garten 1–9, 24118 Kiel, Germany; 2 Department of Biology, Keio University, 4-1-1 Hiyoshi, Yokohama, 223-8521 Japan; 3 Urban Entomology Laboratory, Vector Control Research Unit, School of Biological Sciences, Universiti Sains Malaysia, 11800 Minden, Penang Malaysia; 4 Present Address: Department of Entomology, University of California, Riverside, CA USA

**Keywords:** Zoology, Biomechanics

## Abstract

We unveiled the penile penetration mechanics of two earwig species, *Echinosoma horridum*, whose intromittent organ, termed virga, is extraordinarily long, and *E. denticulatum*, whose virga is conversely short. We characterised configuration, geometry, material and bending stiffness for both virga and spermatheca. The short virga of *E. denticulatum* has a material gradient with the stiffer base, whereas the long virga of *E. horridum* and the spermathecae of both species are homogeneously sclerotised. The long virga of *E. horridum* has a lower bending stiffness than the spermatheca. The virga of *E. denticulatum* is overall less flexible than the spermatheca. We compared our results to a previous study on the penetration mechanics of elongated beetle genitalia. Based on the comparison, we hypothesised that the lower stiffness of the male intromittent organ comparing to the corresponding female structure is a universal prerequisite for the penetration mechanics of the elongated intromittent organ in insects.

## Introduction

The penetration of slender structures without buckling failure is a challenging task from the mechanical point of view, but organisms have overcome this challenge^1,2^. Well-studied examples in insects are steerable probes in variable substrates, such as elongated rostra (snout) of chestnut weevils^3–6^ and ovipositors of wasps^7–9^. In these cases, the penetration process occurs while excavating channels in substrates. Due to the analogy of percutaneous instruments in medicine, unveiling the principles of probes in nature has a potential to be applied to such man-made medical tools^1,2^.

In contrast, penetration of elongated intromittent organs in insects lacks an excavation process. However, these organs seem to be an analogue to rostra and ovipositors. It was, for instance, demonstrated that the intromittent organ of the leaf beetle *Cassida rubiginosa* Müller, 1776 is an elaborate probe, and a potential contribution of the knowledge about its mechanics was previously pointed out for medical tool developments, i.e. catheters^10–12^. There is a common functional prerequisite between those biological probes: It is essential to avoid buckling failure during the penetration process, while flexibility remains mandatory to fit in corresponding structures or to be able to steer in substrates^6,8,9,12^. Nevertheless, in contrast with rostra and ovipositors, the penetration mechanics of the elongated intromittent organ remains mostly unclear.

There are hitherto only a few biomechanical studies on penile penetration of aforementioned cassidine beetles despite the prevalence of the elongated intromittent organ in animals^13,14^. In Cassidinae, males have an elongated intromittent organ, called flagellum, and females also have an elongated spermathecal duct^15,16^. The male flagellum is slender and in some species much longer than body length. During copulation, it is inserted into the spermathecal duct to transfer sperm^15^. The spermathecal duct is often highly coiled, and both the degree of coil curvatures and the shape vary among species^16,17^. Numerical simulations demonstrated that the curvatures of the coils and the presence of reversal turns affect penetration velocities^10^. Moreover, geometry, material distributions and bending stiffnesses of the male and female structures play indispensable roles in the penile penetration^11,12^. The penetration force is applied at its base due to the muscle contraction surrounding the entire flagellum^18^. The cylindrical geometry and the gradient of bending stiffness along the flagellum enable it to penetrate complex-shaped female ducts by fitting the coil shape^12^. In *C. rubiginosa*, a material gradient at the flagellum tip might help to avoid mechanical damage since the spermathecal duct seems to be well-sclerotised and less flexible than the flagellum^11,12^^.^. The generality of these functional principles, i.e. the cylindrical geometry, bending stiffness gradient of the flagellum, and higher bending stiffness of the flagellum comparing to the spermatheca, has not been clarified yet.

In the present study, we aimed to test the generality of the abovementioned functional principles demonstrated and hypothesised based on the cassidine beetle genitalia and addressed the following questions: (1) Are the functional principles limited to the beetles? (2) Are they correlated to the genital length? (3) Do these functional principles depend on the mechanical properties of corresponding female structures? For this purpose, here we chose a distantly related insect taxon, order Dermaptera^19^ and compared their genitalia with the cassidine beetle genitalia. We focused on two pygidicranid earwig species *Echinosoma horridum* Dohrn, 1863, whose intromittent organ, termed virga, is long and narrow^20^, and *E. denticulatum* Hincks, 1959, whose homologous structure is conversely short^21,22^. We characterised their geometry and analysed material distributions within male and female interacting genital structures using imaging and microscopy techniques, e.g. scanning electron microscopy and confocal laser scanning microscopy. The material stiffness (i.e., Young's modulus) was estimated based on micrographs, and resulting data were used to calculate the bending stiffness (i.e., flexural rigidity) of the virga and spermatheca. Based on results, we determine the general principles of the penetration mechanics of the genitalia in earwigs and compare them with the previous studies on the beetle genital.

## Results

### Morphology of genitalia

**Figure 1:**
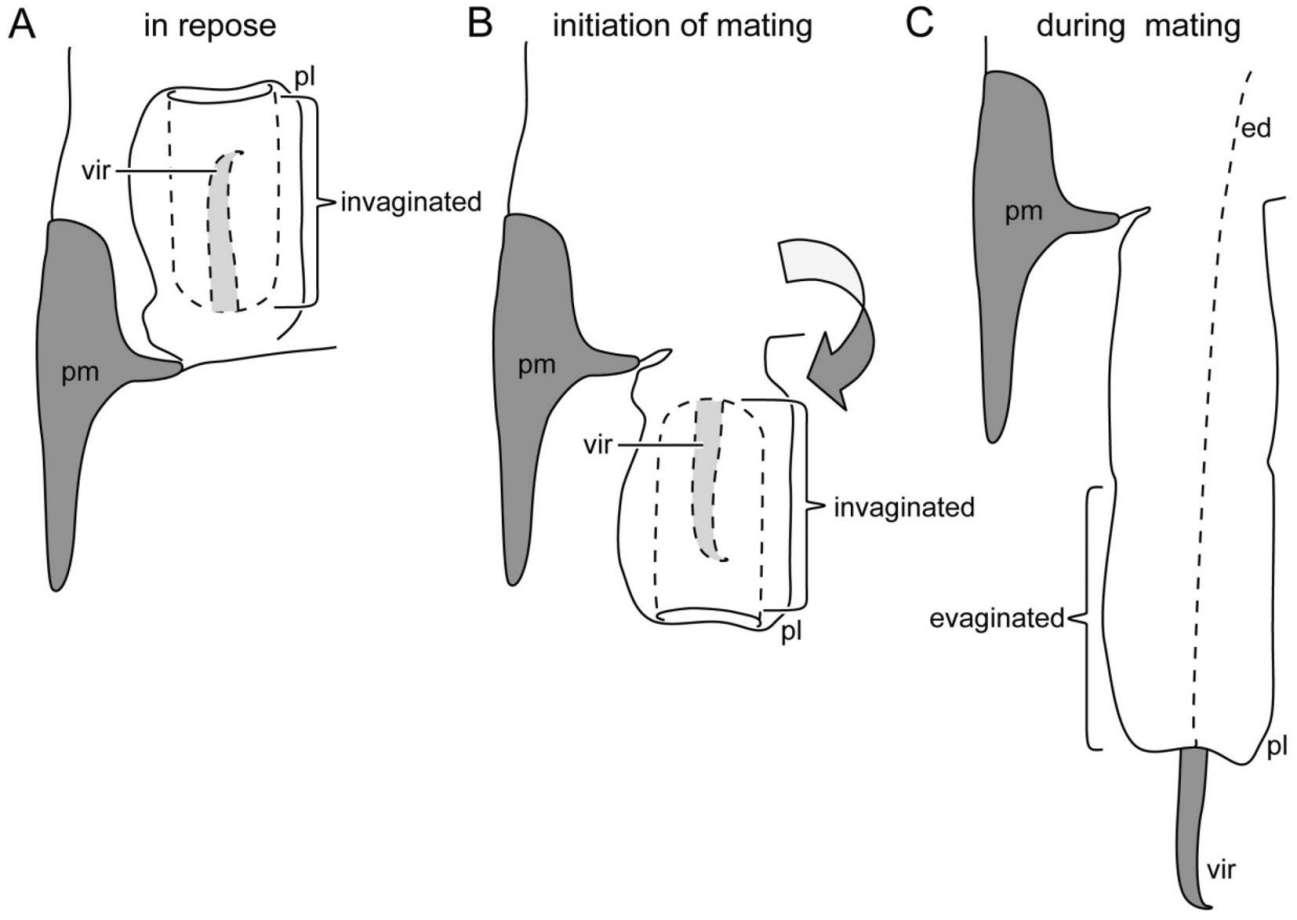
Schemes of the male genitalia of Pygidicranidae. The left half is drawn. (**A**) The virga and the posterior half of the penis lobe are invaginated in its anterior half, and the entire penis lobe orients cranially in repose. At the initiation of mating, the penis lobe is unfolded and orients caudally in the female genital chamber (**B**), and the invaginated part of the penis lobe and the virga are evaginated and penetrate in the female genital chamber (**C**). Abbreviations: ed, ejaculatory duct; pl, penis lobe; pm, paramere; vir, virga.

As a plesiomorphic characteristic of the extant earwigs, males of *Echinosoma* species have paired penis lobes, each of which encloses a rod- or filament-like virga that orients cranially in repose (Fig. 1A)^20,23^. Females of *Echinosoma* have a single, tube-shaped sperm storage organ, termed spermatheca^21,24^. In most earwig species, one of the paired penis lobes is unfolded and inserted into the female vagina at the initiation of mating, and the virga is inserted into the spermatheca of a female and functions as a sperm-transferring tube during copulation (Fig. 1B,C)^25^.

**Figure 2:**
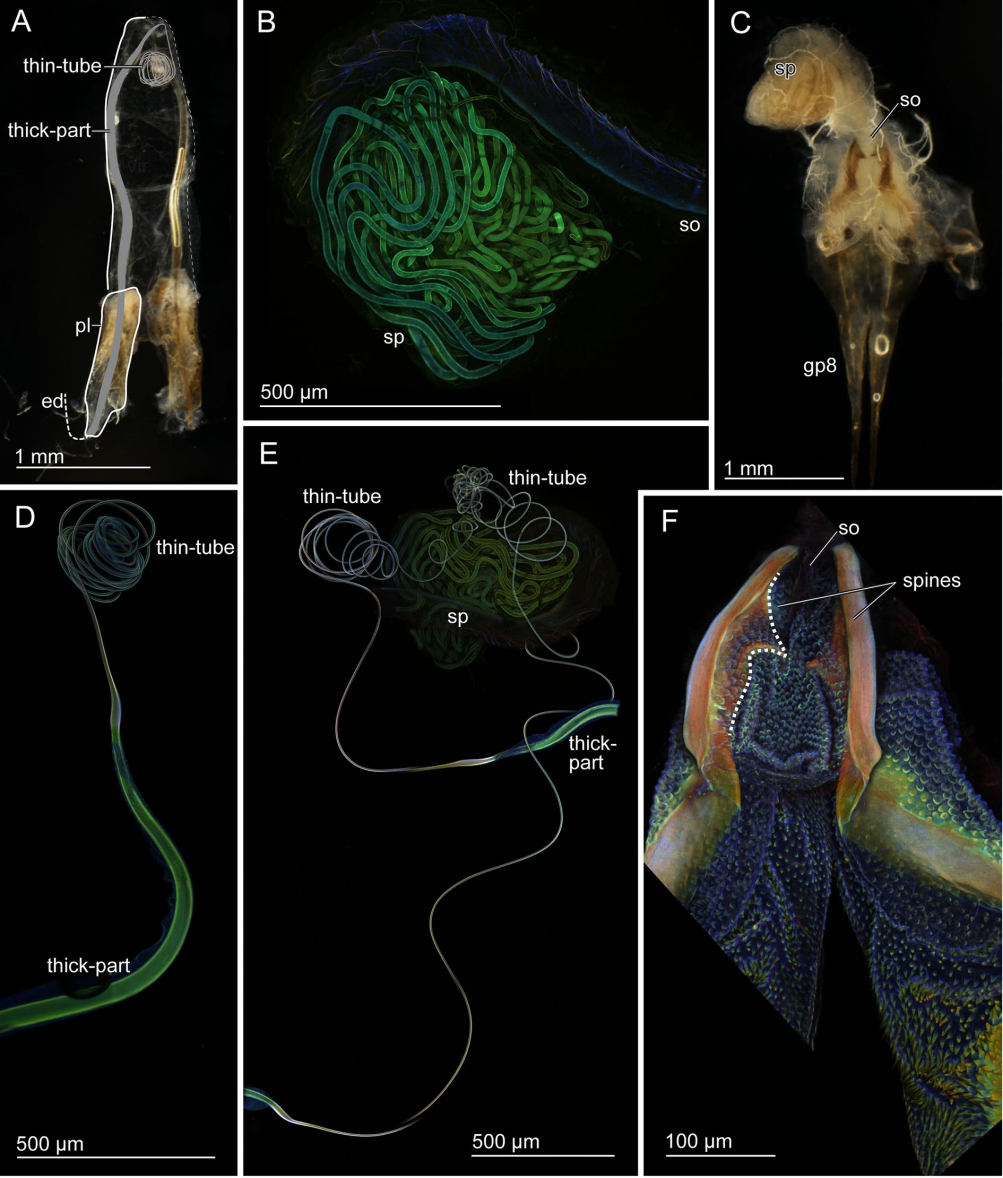
Male and female genitalia of *Echinosoma horridum*. (**A**, **C**) Stereomicrographs, (**B**, **D**–**F**) confocal laser scanning micrographs. (**A**) Male genitalia. Paired virgae are situated in the genitalia chamber and directed cranially in repose. Half of the paired structures is highlighted. (**B**) Spermatheca. (**C**) Female genitalia. (**D**) Virga. (**E**) Virgae and spermatheca. (**F**) A pair of spines in the vicinity of a spermatheca opening. Abbreviations: ed, ejaculatory duct; gp8, gonapophysis VIII; pl, penis lobe; so, spermatheca opening; sp, spermatheca; vir-thick, thick-part of virga; vir-thin, thin-tube of virga.

In *E. horridum*, males have extremely long paired virgae, which are almost as long as their body length (Table 1). The virga is composed of a thicker base, which tapers towards the apex and a long thin tube, which coils in the genital chamber when the virga is not in use (*thick-part* and *thin-tube*, Fig. 2A). Their transition zone tappers sharply and appears to be structured (Fig. 4A,B), as if the gonopore was situated, but our observation revealed that the virga, from the thicker base to the distal end of the thin-tube, is an entire tube with no opening in this zone (Fig. 4C–I). The penis lobe of *E. horridum* males does not carry any spiny auxiliary sclerites, unlike that of *E. denticulatum*. In the female, the spermatheca is situated on the dorsal side of the vagina (Fig. 2C) and is a convoluted tube (Fig. 2B). The spermatheca of *E. horridum* is more than four times longer than the insect body length (Table 1). Paired spines are present and lay adjacent to the spermathecal opening (Fig. 2F).

**Table 1:** Measurements of the body length and genitalia of *Echinosoma horridum* and *E. denticulatum*, mean ± sd. Forceps lengths were included in the body size measurements. The numbers in the parentheses represent the number of individuals used for measuring the lengths.

	Male		Female	
	Body length (mm)	Virga length (mm)	Body length (mm)	Spermatheca length (mm)
*Echinosoma horridum*	12.66 ± 0.19 (4)	10.77 ± 1.02 (5)	11.89 ± 0.47 (3)	45.84 ± 2.20 (2)
*Echinosoma denticulatum*	12.71 ± 0.28 (3)	0.58 ± 0.018 (4)	12.79 ± 2.02 (3)	8.46 (1)

**Figure 3:**
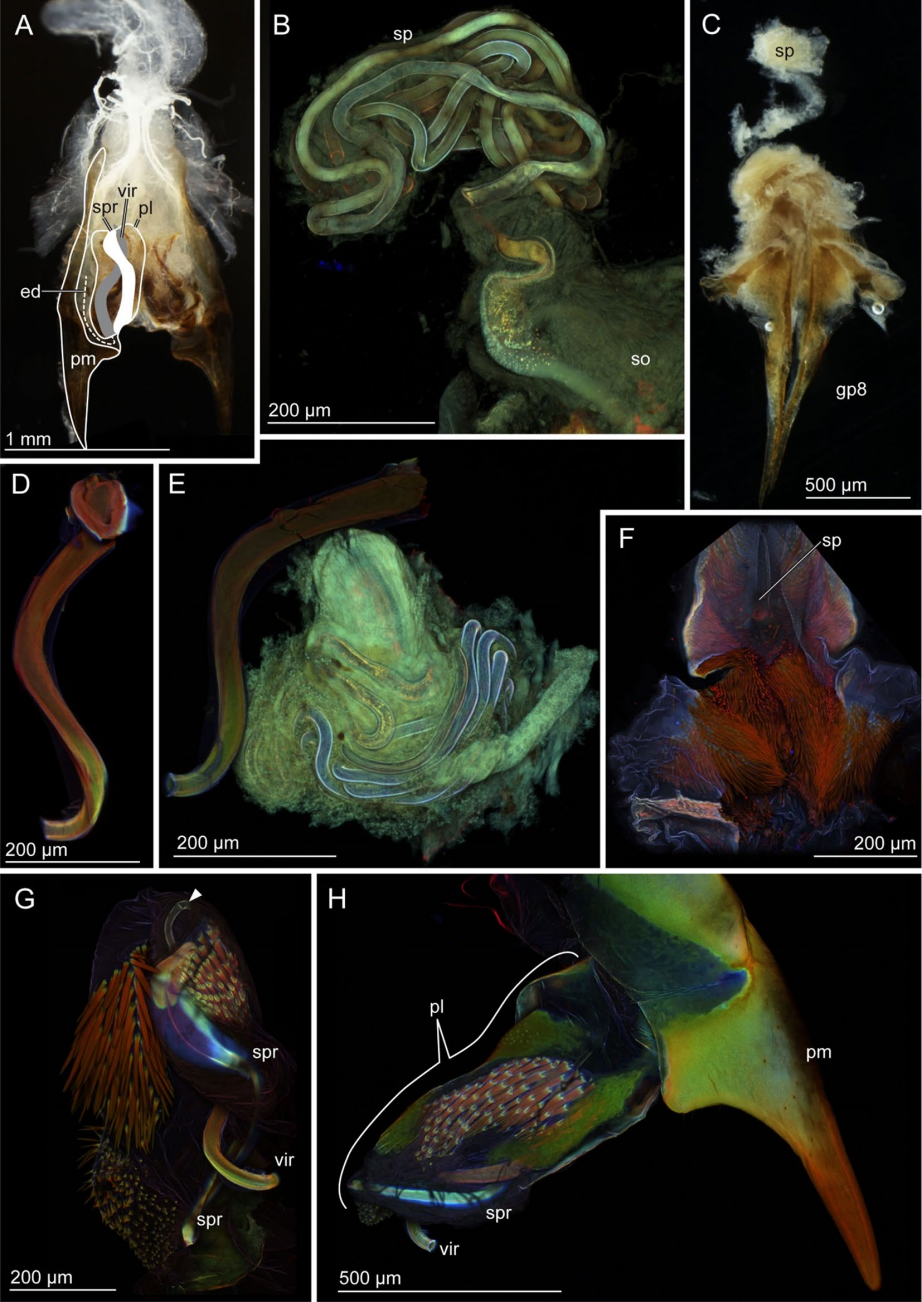
Male and female genitalia of *Echinosoma denticulatum.* (**A**, **C**) Stereomicrographs, (**B**, **D**–**H**) confocal laser scanning micrographs. (**A**) Male genitalia. Paired penises are situated in the genitalia chamber and direct cranially in repose as seen here. Half of the paired structures is highlighted. (**B**) Spermatheca. (**C**) Female genitalia. (**D**) Virga. (**E**) Virga and spermatheca. (**F**) Numerous setae surrounding a spermatheca opening. (**G**) Virga and its surrounding penis lobe carrying a resilin-enriched spring and numerous spines. The arrowhead denotes the opening of the ejaculatory duct. (**H**) Paramere, virga and penis lobe. Abbreviations: ed, ejaculatory duct; gp8, gonapophysis VIII; pl, penis lobe; so, spermatheca opening; sp, spermatheca; spr, spring; vir, virga.

Males of *E. denticulatum* possess very short paired virgae (Fig. 3A, Table 1), carrying a radially expanding brim at the tip (Fig. 6A,B). The invaginated surfaces of the penis lobes are covered with dozens of spines (Fig. 3G,H). The penis lobe encloses a helicoidal virga (Fig. 3D) and an associated sclerite, termed spring herein, due to the resilin richness as described below (see material distributions). The spring is also helicoidal and turns in the opposite direction, forming a seemingly double-helix structure with the virga (see *vir* and *spr* in Fig. 3G,H). Females carry hundreds of long setae at the spermathecal opening (Fig. 3F). The length of the spermatheca of *E. denticulatum* is two-thirds of the body length (Table 1), and the intact spermatheca is a convoluted tube (Fig. 3B,C).

### The geometry of virgae and spermathecae

To examine the geometry, we cut specimens into either three (base, middle, apex) or four (thick-part, thin base, thin middle, thin apex in the *E. horridum* male) regions, observed their cross-sections and measured the diameter and wall thickness. Due to the difficulty in sample preparation, we measured these parameters as many as we could from every individual. We calculated the measurements described below based on all measured data and performed statistical tests using means of the measurements for each individual to avoid pseudoreplication. Detailed measurement data is available in supplementary Tables 1–6.

**Figure 4:**
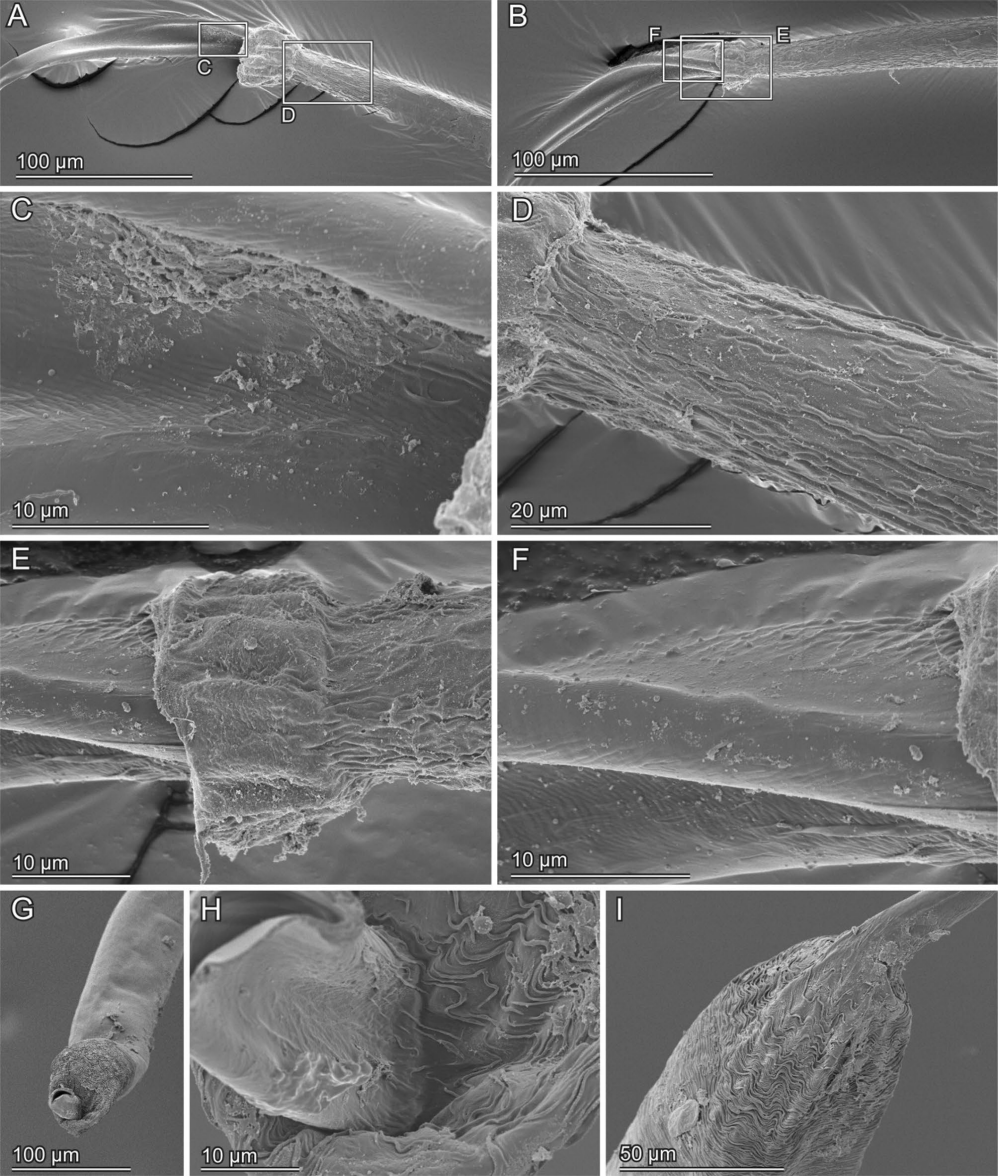
Scanning electron micrographs of the transition zone of the virga of *Echinosoma horridum.* (**A**) Overview. The inner side. (**B**) Overview. The outer side. (**D**–**F**) Magnified surfaces marked in images (**A**) and (**B**). (**G**–**I**) Caudal views of the transition area between the thick-part and thin-tube of the virga.

**Figure 5:**
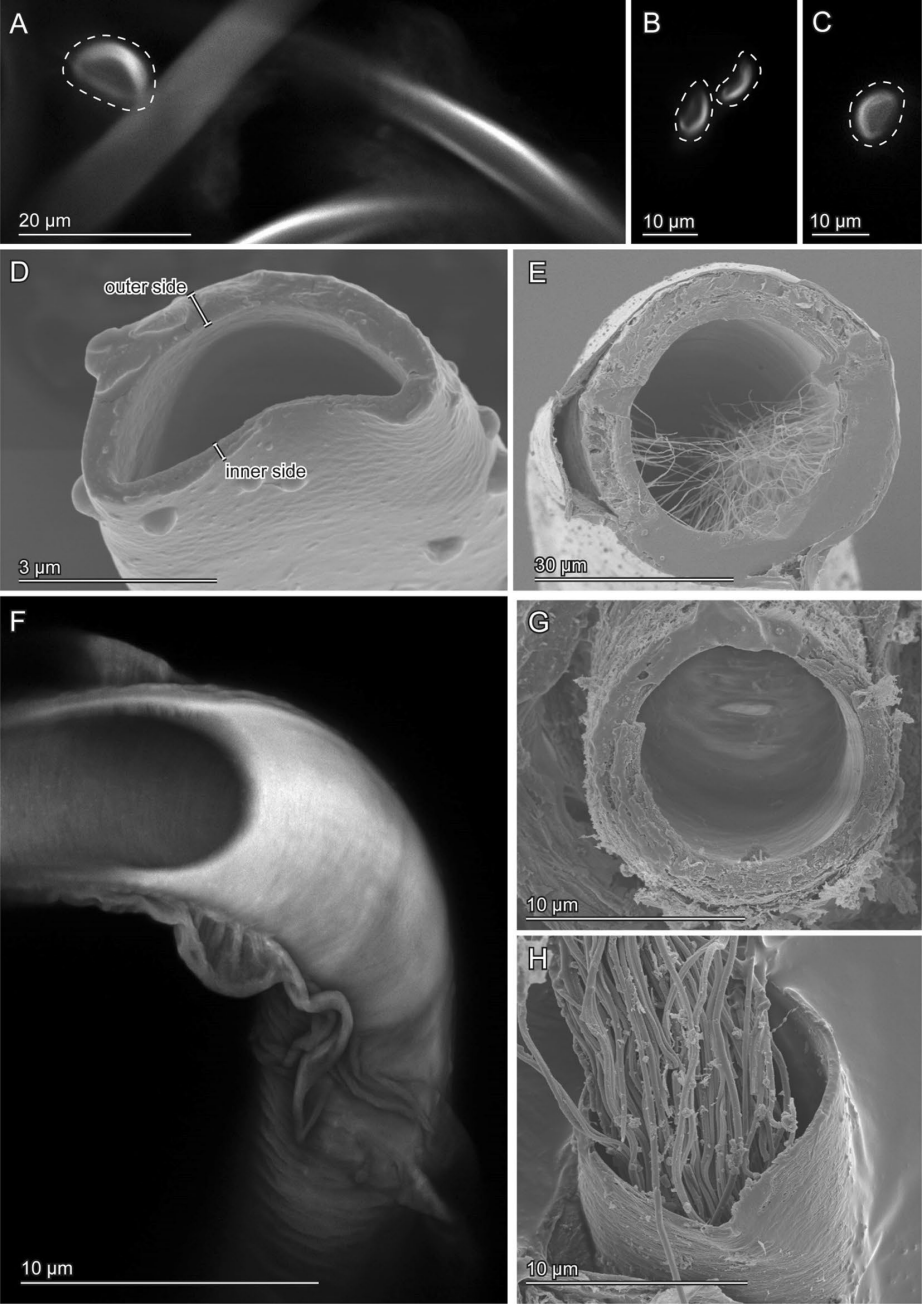
Geometry of the virga and spermatheca of *Echinosoma horridum*. Confocal laser scanning micrographs (**A**–**C**,**F**) and scanning electron micrographs (**D**, **E**, **G**, **H**). (**A**–**C**) Optical slices of the thin-tube of the virga taken using CLSM. Cross-sections are enclosed with broken lines. (**D**) A cross-section of the thin-tube of the virga. The cross-section was visualised by cutting the thin-tube with micro scissors that caused slight deformation (cf. images **A**–**C**). (**E**) A cross-section of the thick-part of the virga. Microtrichia in the lumen are visible. (**F**) The thick-part of the virga. A maximum intensity projection. A distal part is optically cut so that the hollow inside is also visible. (**G**, **H**) Cross-sections of the spermatheca. Spermatozoa are visible (**H**).

The cross-sections of the virga in *E. horridum* was circular in the thick-part (Fig. 5E,F). The virga abruptly flattened in the transition zone between the thick-part and the thin-tube (Fig. 4A,B). The internal surface of the thick-part partly carried microtrichia (Fig. 5E). The entire thin-tube was hollow and was flattened semi-circular to crescent-like in cross-section (Fig. 5A–D). The diameter of the thick-part was 43.20 ± 7.87 µm (*N* = 11) [mean ± sd], and the wall thickness of the same location was 6.28 ± 1.30 µm (*N* = 61) (Fig. 7A,C,E). The thin-tube had a diameter of 5.74 ± 1.23 µm (*N* = 12) (Fig. 7A), which was calculated from circumferences with an assumption that the cross-section is annular. The wall thickness was not constant within a circumference; the outer side of the circumference had a thicker wall comparing with that of the inner side [0.60 ± 0.20 µm (*N* = 27) vs 0.26 ± 0.10 µm (*N* = 24)] (Figs. 5A–D, 7C). Among the measurement positions of the thin-tube, the diameter (Kendall’s rank correlation coefficient τ = − 0.467, *N* = 7, *P* = 0.1742), the wall thicknesses of the outer (*τ* = − 0.281, *N* = 11, *P* = 0.264) and the inner side (τ = − 0.185, *N* = 11, *P* = 0.457) were statistically consistent with neither increasing nor decreasing trend.

**Figure 6:**
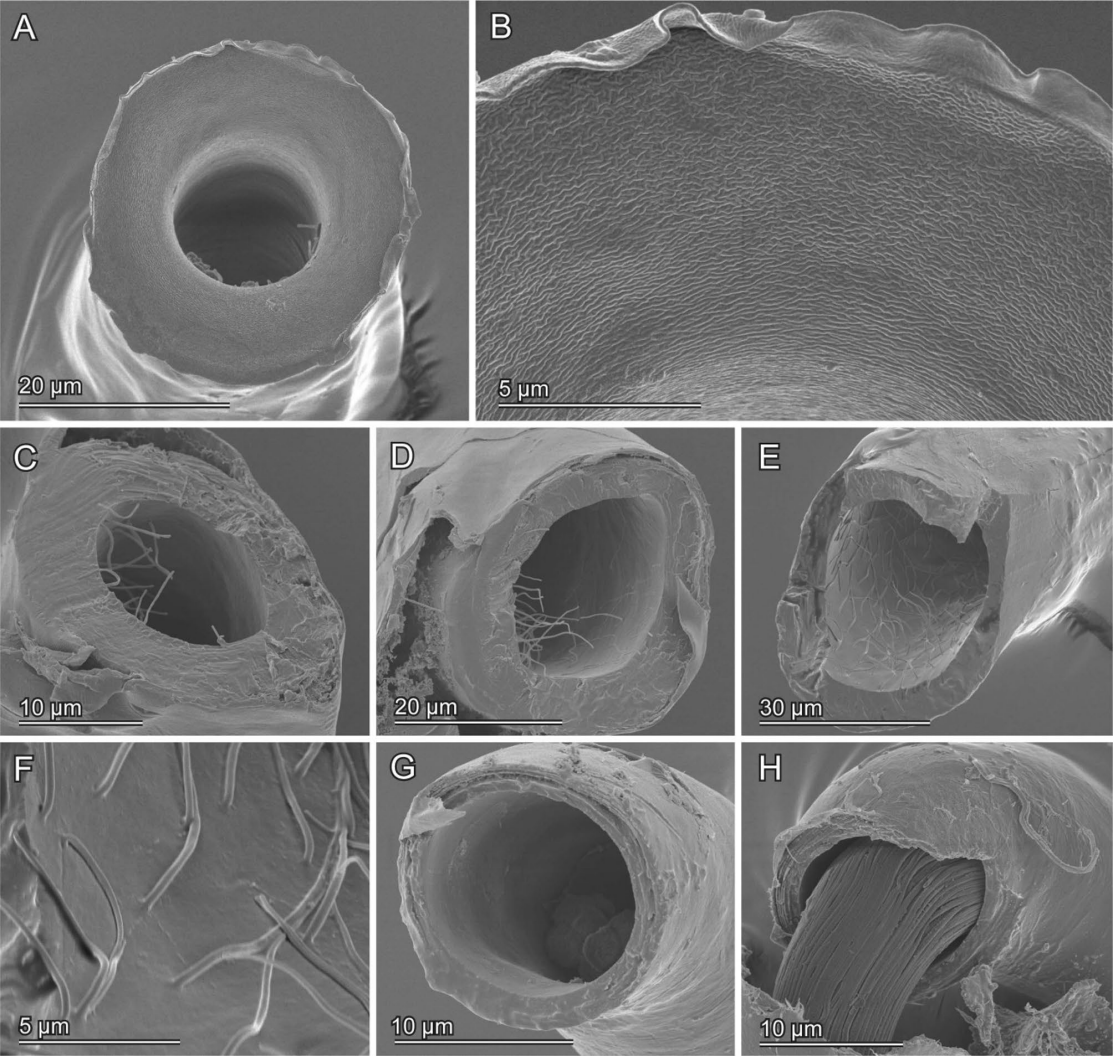
Geometry of the virga and spermatheca of *Echinosom denticulatum*. Scanning electron micrographs of the virga (**A**–**F**) and the spermatheca (**G**, **H**). (**A**) The very tip of the virga. (**B**) A magnified image of the tip of the virga. (**C**–**F**) Cross-sections of the virga at the apex (**C**), at the middle (**D**) and at the base (**E**). (**F**) A magnified surface images of the inner surface of the virga. (**G**, **H**) Cross-sections of the spermatheca. Spermatozoa are visible (**H**).

**Figure 7:**
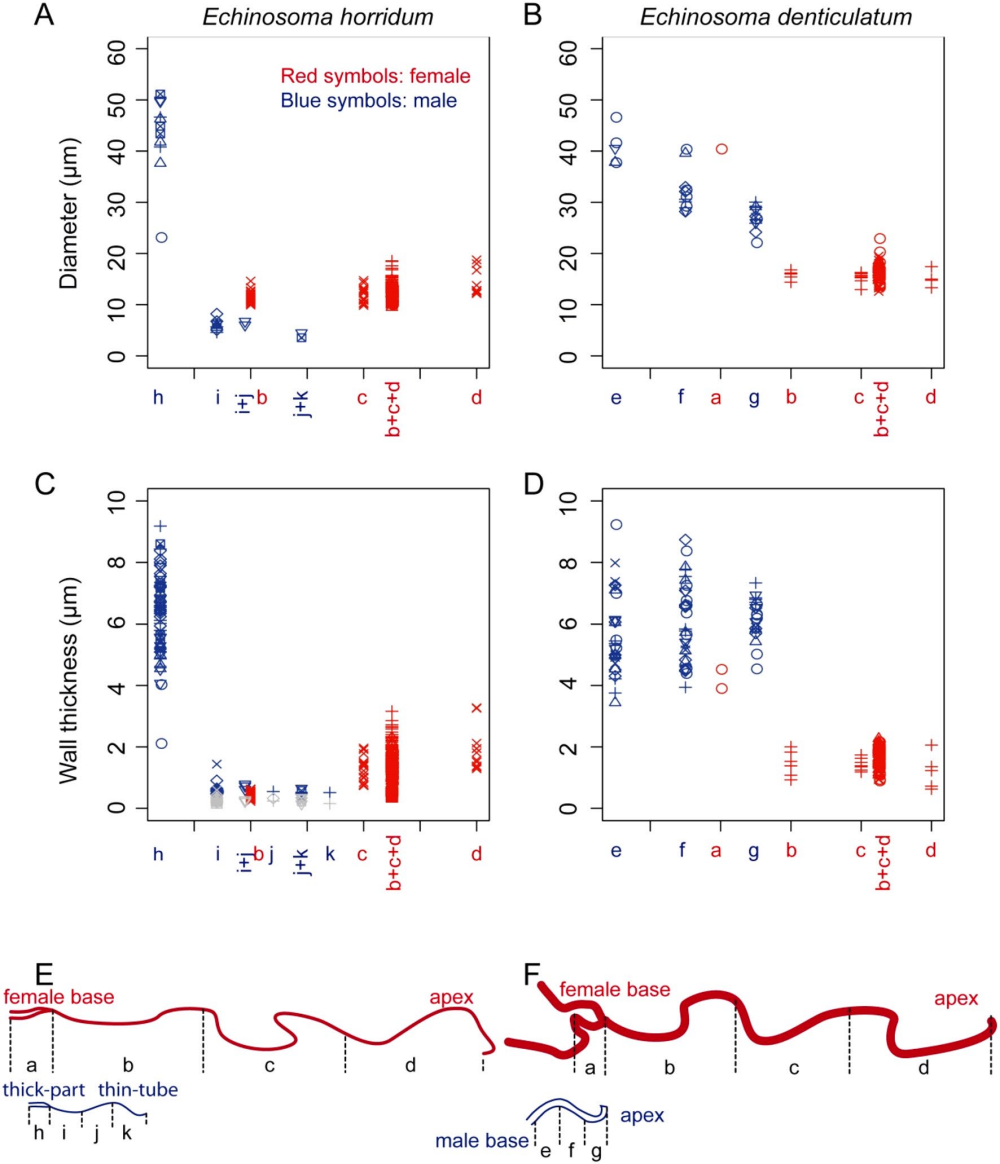
Geometry of the virga and spermatheca of *Echinosoma horridum* (**A**, **C**, **E**) and *E. denticulatum* (**B**, **D**, **F**). (**A**, **B**) Outer diameters of the virga and spermatheca. (**C**, **D**) Wall thicknesses of the virga and spermatheca. These were not constant along circumferences in the thin-tube of the virga of *E. horridum*, and larger wall thicknesses and smaller ones are shown respectively with blue and grey colouration in (**C**). Each symbol in (**A**–**D**) represents data from one individual. The letters correspond to locations, whose diameters were measured and shown in schemes of the virga and spermatheca (**E**, **F**). Since it was impossible to measure wall thicknesses and diameters for each area separately, we needed to categorise some areas together, i.e. i + j, j + k and b + c + d. Physically interacting areas between the virga and spermatheca during mating are depicted as overlapped each other in every graph.

The cross-section of the virga in *E. denticulatum* was approximately circular (Fig. 6). The virga tapered from the base to the apex [40.83 ± 3.62 µm (*N* = 5) in the base, 32.23 ± 3.92 µm (*N* = 12) in the middle, 26.91 ± 2.36 µm (*N* = 11) in the apex] (*τ* = − 0.779, *N* = 14, *P* < 0.001) (Fig. 7B,F). The wall thickness of the virga was constant along the measurement locations (*τ* = 0.233, *N* = 18, *P* = 0.225) and was 5.94 ± 1.19 µm (*N* = 81) (Fig. 7D,F).

The spermatheca geometry of both species was simpler than the male ones. In both species, it showed a circular cross-section (Figs. 5G,H, 6G,H). In *E. horridum*, the diameter was constant among the measurement locations [12.66 ± 1.72 µm (*N* = 158)] (*τ* = 0.258, *N* = 6, *P* = 0.499) (Fig. 7A,E), and the wall thickness slightly increased from the base to the apex [0.44 ± 0.094 µm (N = 45) in the base, 1.25 ± 0.39 µm (N = 19) in the middle, 1.91 ± 0.66 µm (*N* = 13) in the apex] (τ = 0.775, *N* = 6, *P* = 0.042) (Fig. 7C,E). In *E. denticulatum*, the diameter was constant among the measurement locations except for the widened basal part [16.20 ± 1.68 µm (*N* = 70)] (τ = − 0.430, *N* = 6, *P* = 0.260) (Fig. 7A,B). The wall thickness of *E. denticulatum* was also constant along the spermatheca [1.71 ± 1.49 µm (*N* = 122)] (τ = − 0.120, *N* = 5, *P* = 0.782) (Fig. 7D,F).

### Material distributions

To analyse material distributions within the virga and spermatheca in the study species, we applied the method established by Michels and Gorb^26^ using confocal laser scanning microscopy (CLSM). We visualised autofluorescence of materials comprising the cuticle with four sets of lasers and filters and assigned blue, green, red (50% saturation) or red (50% saturation) to each image produced with each set of lasers and filters, respectively. We interpreted resulting overlaid images as follows: (1) red-coloured structures are well-sclerotised and stiff, (2) green coloured ones are less sclerotised and (3) blue-coloured ones are composed of the larger amount of resilin and are likely softer than the other parts.

Heterogeneity of a material distribution along the virga was detected in the virga of *E. horridum*, but it was not remarkable (Fig. 2D, supplementary Fig. 1). It tended that the thick-part of the virga was visualised with green and that the thin-part was whitish-blue. The transition zone between the thick-part and thin-tube was visualised with white, i.e. a mixture of three primary colours. The thick-part is likely moderately sclerotised, while the thin-part is less sclerotised comparing to the thick-part of the virga within each individual. The transition zone may be more sclerotised than the rest. Among the four virgae used for the CLSM analysis, whose autofluorescence intensities were individually adjusted, there was one exceptional case where the thin-tube was visualised with reddish colouration. However, the thick-part and transition zone were comparable to the results mentioned above (supplementary Fig. 1).

The spermatheca of *E. horridum* seems to be moderately sclerotised (green to reddish-green), and no gradients were observed except for the proximal part, plausibly membranous (dark blue) (Fig. 2B, supplementary Fig. 2). Comparisons between the sexes of *E. horridum* (Fig. 2E, supplementary Fig. 3) showed that the virga and spermatheca were green to white, indicating that sclerotised levels of these structures are comparable to each other.

A material gradient was observed along the virga of *E. denticulatum* (Fig. 3D, supplementary Fig. 4). The virga has a well sclerotised base and middle, which were visualized in red, and possesses a less sclerotized apical part (Fig. 3D, supplementary Fig. 4).

The spermatheca of *E. denticulatum* was visualised homogeneously with bluish-green, indicating that it is likely less sclerotised (Fig. 3B, supplementary Fig. 5). Comparative results were also obtained from micrographs of male and female structures recorded together (Fig. 3E, supplementary Fig. 6). These images suggest that the spermatheca is likely less sclerotised in comparison to the virga but the very tip of the virga.

We analysed autofluorescence of some other genital structures as well, i.e. genital spines and thick setae (Figs. 2F, 3F–H). The hundreds of penile spines and long female setae of *E. denticulatum* and the paired spines of the *E. horridum* female were red in all the analysed conditions (Figs. 2F, 3F–H). It seems that those long setae and spines are well-sclerotised. Another remarkable result is that the sclerite situated at the penis lobe of *E. denticulatum* is seemingly resilin dominated, which here is termed spring (Fig. 3G,H).

### Bending stiffness (flexural rigidity, *EI*)

Bending stiffness is defined as the product of Young's modulus (*E*) and the second moment of area (*I*). Bending stiffness of the virga and spermatheca was estimated using the calculated second moment of area based on either SEM or CLSM images using the BoneJ plugin^27^ for Fiji and the estimated Young's modulus based on the CLSM images using the program developed by Eshghi et al.^28^.

**Figure 8:**
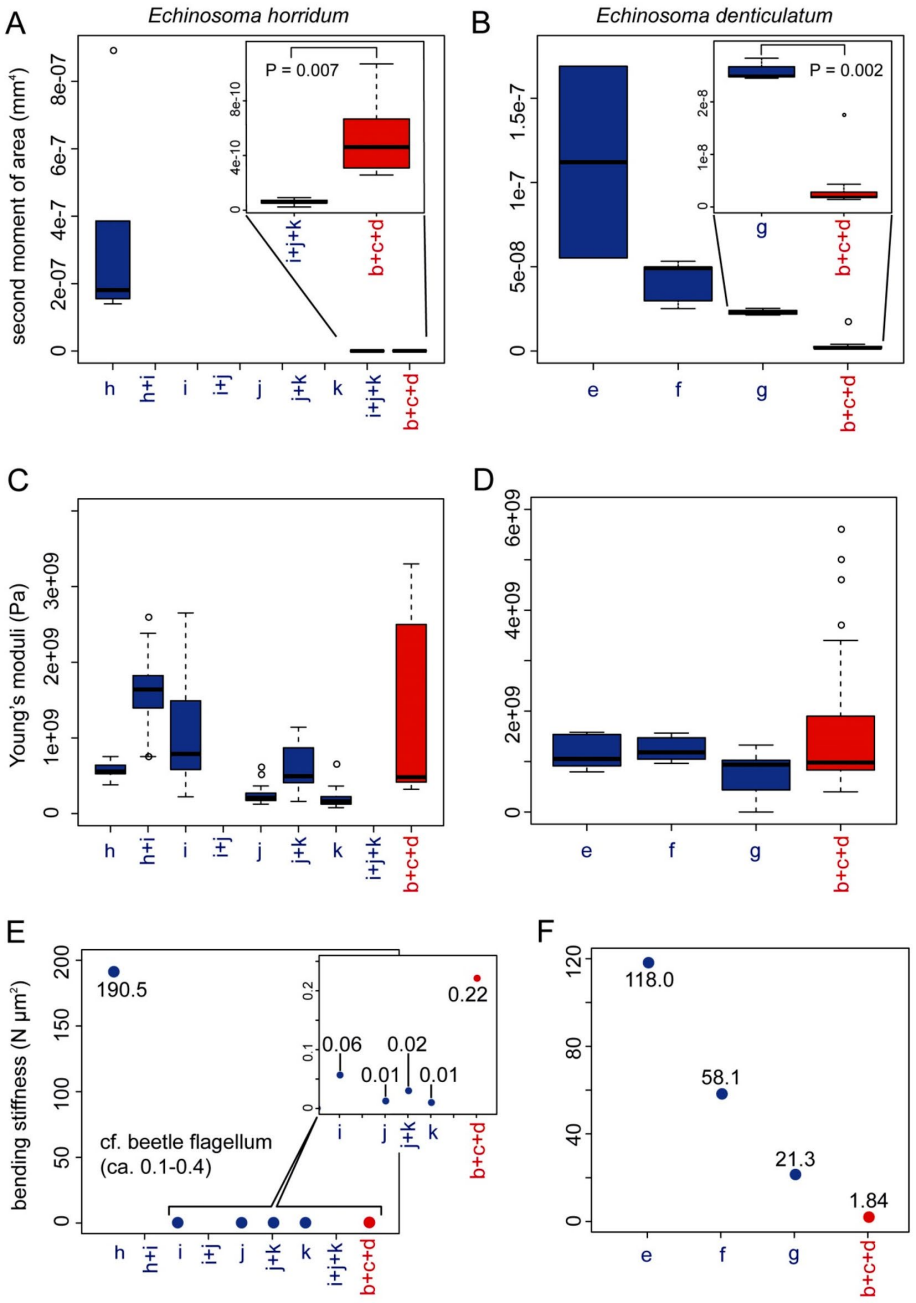
The second moment of area, Young's moduli and bending stiffnesses along the virga and spermatheca of *Echinosoma horridum* (**A**, **C**, **E**) and *E. denticulatum*. (**B**, **D**, **F**). (**A**, **B**) The calculated second moment of area using Fiji equipped with the plugin BoneJ. (**C**, **D**) Estimated Young's moduli. (**E**, **F**) Resulting bending stiffnesses. The alphabets correspond to locations whose second moment of area and Young's modulus were measured and shown in schemes of the virga and spermatheca (**E**) and (**F**) in Fig. 7.

The second moment of area, i.e. a measurement of structural stiffness results from a cross-sectional shape, was calculated based on the SEM or CLSM images, in which cross-sections were neither deformed nor tilted. Dried samples that were prepared for the SEM were brittle and could simply be broken (i.e. no plastic deformation), except for the thin-tube of the virga of *E. horridum*, which was impossible to fracture. Hence, we had to cut them using a blade and micro-scissors, which could have resulted in permanent deformation of cross-sections (see Fig. 5A–C vs Fig. 5D, which shows the least deformation among SEM images we have taken). Therefore, for the thin-tube of the virga of *E. horridum*, we used optical slices of CLSM images (Fig. 5A–C), but it was impossible to recognise the base, middle and apex of the thin-tube with this method (Fig. 8A).

In *E. horridum*, the second moment of area was more than three orders of magnitude higher in the thick-part of the virga (1.81 × 10^–7^ mm^4^, hereafter we present medians and used them for calculation of the bending stiffness, due to high variations in estimated Young's moduli presented below) than that of the thin part (6.02 × 10^–11^ mm^4^). The second moment of area of the spermatheca (4.61 × 10^–10^ mm^4^) was statistically higher than that of the thin-part of the virga (Mood's median test: *N*_male_ = 6, *N*_female_ = 15, *P* = 0.0070) (Fig. 8A). This result corresponded to the abovementioned geometric data showing that the diameter and the wall thickness of the spermatheca of *E. horridum* were smaller than those of the thick-part of the virga and larger than those of its thin-tube (Fig. 7A,C). To check the validity of the data, we calculated the second moment of area manually based on the abovementioned geometric measurements (see the formula in materials and methods), and the manually calculated values were comparable to these ones.

The second moment of area of the virga in *E. denticulatum* decreased from the base to the apex (1.12 × 10^–7^ mm^4^ in the base, 4.91 × 10^–8^ mm^4^ in the middle and 2.27 × 10^–8^ mm^4^ in the apex; τ = − 0.744, *N* = 11, *P* = 0.0039; Fig. 8B). The corresponding value of the spermatheca except for the widened basal region was statistically smaller (1.88 × 10^–9^ mm^4^) than that of the virga in the apical region (Wilcoxon rank-sum test: *W* = 40, *N*_male_ = 4, *N*_female_ = 10, *P* = 0.0020) (Fig. 8B). This difference was also visible in the geometric data (Fig. 7B). The manually calculated second moment of area was comparable to the calculations.

Estimated Young's moduli varied much within each measurement location (Fig. 8C,D). The estimated Young’s moduli showed neither increasing nor decreasing tendency along the measurement locations in the male (*E. horridum*: the thick-part: 0.56 GPa; the transition zone between the thick-part and thin-tube: 1.64 GPa, the thin-tube: 0.16–0.79 GPa; τ = − 0.744, *N* = 7 , *P* = 0.244; Fig. 8C) (*E. denticulatum*, the base: 1.05 GPa; the middle: 1.18 GPa, the apex: 0.94 GPa; τ = − 0.415, *N* = 6, *P* = 0.164; Fig. 8D). However, the sclerotisation level of the transition zone of the virga in *E. horridum* may be slightly higher than the rest of the virga [τ = − 0.415, *N* = 8, *P* = 0.164 (the thick-part was removed from the statistical test); Fig. 8C], and this corresponded to the CLSM results described above. Although it was not statistically significant, in *E. denticulatum*, the apical part of the virga showed a slightly lower Young's modulus than that of the rest, as expected based on the CLSM results described above. The Young's moduli in the female except for the widened basal region were estimated without any information on measurement locations, and all data were pooled for each species (*E. horridum*: 0.48 GPa, *E. denticulatum*: 0.98 GPa).

Based on those results (the Young's modulus and second moment of area), we calculated bending stiffnesses of the virga and spermatheca (Fig. 8 E, F). Since there was no significant difference in the estimated Young's moduli among the measurement locations, the results resembled those of the second moment of area. In *E. horridum*, the thick-part of the virga showed the highest bending stiffness (190.5 N µm^2^) followed by the spermatheca (0.22 N µm^2^) and the thin-tube (0.01–0.06 N µm^2^) (Fig. 8E). Since it was not possible to calculate the second moment of area at each measurement location with our method (Fig. 8A), the sole $I$ value calculated in the study was applied to all the estimated Young's moduli of the thin-tube of *E. horridum* (Fig. 8C,E). The bending stiffness of *E. denticulatum* was overall higher in the male than that in the female (Fig. 8F). In the basal and middle regions of the virga (118.0 N µm^2^ in the base and 58.1 N µm^2^ in the middle), the bending stiffnesses were higher than that of the apical region (21.3 N µm^2^) and that of the spermatheca except for the basal widened region (1.84 N µm^2^) (Fig. 8F).

## Discussion

The present study shows that males and females of the pygidicranid earwig *E. horridum* have extremely long genitalia (85.1% of body length in the male and 386% in the female). In contrast, in the other earwig species *E. denticulatum*, the genitalia are much shorter (4.5% of body length in the male and 66.1% in the female). Despite the tremendous difference in genital size, the genital configuration is common between the study species: Male external genitalia are composed of paired penis lobes with virgae situated in the genital chamber with the position directing cranially (Fig. 1A), and females have one spermatheca. This configuration of male and female genitalia is commonly known in Pygidicranidae^21,25^. Male genitalia with paired penis lobes are plausibly plesiomorphic conditions in Dermaptera^29,30^, although a plesiomorphic state of the orientation of the penises in repose remained unclear^30^.

Kamimura and Lee^21^ examined genital coupling of *E. denticulatum* and reported that one of the paired penis lobes is extended caudally and evaginates during mating (Fig. 1B,C). The virga is then inserted into the female vagina and the wide proximal region of the spermatheca for transferring sperm. The penis lobe functions as a storage space of the virga and spiny area in repose^21^. In the present study, we found a resilin-enriched and helicoidal spring forming a double-helix structure with the helicoidal virga (Fig. 3G,H). The spring likely assists the virga to quickly move back and forth while the penis lobe is evaginated by increased haemolymph pressure and is pulled back.

Moreover, except for the basal widened region where is plausibly membranous, the spermatheca is narrower than the virga (Fig. 7B). This implies that the tip of the virga, carrying a radially expanding brim, can fit the entrance of the thin spermathecal duct and avoid flowing sperm backward during insemination. At the same time, the tip of the short virga may encounter the highest risk of breakage during the insertion process since the tip has frequent physical contacts while exposed to the female genital chamber. Therefore, we expect that the bending stiffness gradients found in the virga of *E. denticulatum*, particularly the less sclerotised and flexible apical part, reduce the contact stresses at this critical zone.

Similar bending stiffness and material gradient were previously reported for the intromittent organ of *Cassida rubiginosa* (Table 2)^11,12^. In this species, the flexibility of the elongated intromittent organ is possibly essential to fit into coils of the spermathecal duct. It is worthwhile to mention that the similar bending stiffness gradients plausibly function as assistances of the variable penetration processes in distantly related insect taxa. The material gradient at the tip of *C. rubiginosa* presumably avoids its breakage^12^, as it is also hypothesised in the present study for *E. denticulatum*. Although the length of the virga of *E. denticulatum* is much shorter than that of *E. horridum* and *C. rubiginosa*, the penile penetration mechanism is fundamentally the same between *E. denticulatum* and *C. rubiginosa*, i.e. the elongated intromittent organ is merely pushed from its base into the sclerotised spermatheca (Table 2)^18^. This commonality between the distantly related species suggests that the presence of material heterogeneity at the apical or tip region is likely related to its penetration mechanism, and this working hypothesis has to be further tested.

**Table 2:** A **c**omparison of the penetration mechanics between *Echinosoma* genitalia and *Cassida rubiginosa* genitalia documented and hypothesised in the present and previous studies.

Species	Male intromittent organ		Interaction between the sexes		Spermatheca	
	Relative length to body length	Bending stiffness gradient	Material distribution heterogeneity	Penile penetration mechanism	Flexibility, comparing to the spermatheca	Shape
*Echinosoma denticulatum*	4.56% (0.58 mm)^a^	Present, apical region more flexible^a^	Present, but not prominent^a^	Pushed from the base^a,e^	Smaller in the male^a^	Convoluted^a,e^
*Echinosoma horridum*	85.1% (10.8 mm)^a^	No gradient, constant along the thin-tube^a^	Absent in the thin-tube^a^	The thin-tube gradually penetrates a folded site, not from the tip^a^ (a hypothesis, Fig. 9)	Larger in the male (the thin-tube of the virga vs spermatheca)^a^	Convoluted^a^
*Cassida rubiginosa*	157% of body length (10.2 mm)^b^	Present, apical region more flexible^c^	Present at the tip^c,d^	Pushed from the base^f^	Larger in the male^b,g^	Regularly coiled^f,h^
Current explanations (hypothesis)	-	Not mandatory for penetration of the elongated penis, and plausibly reduces the risk of penile breakage	Plausibly reduces the risk of penile breakage	-	Universal prerequisite for penetration of the elongated penis	-

^a^The present study, ^b^Matsumura et al.^36^, ^c^Matsumura et al.^12^, ^d^Filippov et al.^11^, ^e^ Kamimura and Lee^21^, ^f^Matsumura et al.^18^, ^g^ Matsumura et al. (submitted), ^h^ Filippov et al.^10^.

In contrast to *E. denticulatum*, information on the reproductive biology in *E. horridum* is hitherto limited. However, since the configuration of the male and female genitalia are congruent between the studied species except for the absence of a spring on the penis lobe in *E. horridum*, the penetration mechanism of the virga may be very alike. In other words, increased haemolymph pressure in one of two penis lobes, carrying the slender virga, causes posterior extension of the penis lobe, evaginating and pushing the virga into the spermatheca (Fig. 1). Contrary to *E. denticulatum*, our geometric data showed that the diameter of the thin-tube in *E. horridum* is small enough to deeply penetrate the spermatheca (Fig. 7A), which implies that the thin-tube of the virga of *E. horridum* can be fully inserted into the spermatheca. The present study also documented that the slender virga is an entire tube continuing from the gonopore, indicating that the male uses the virga to transfer sperm, as in *E. denticulatum*. Moreover, the spermatheca of *E. horridum* is much longer than that of *E. denticulatum*. Such a positive correlation between the virga and spermatheca lengths connotes that the long virga has physical interactions with the spermatheca since such a correlation between elongated intromittent organs and corresponding spermathecae are well-known in insects^15,31–33^.

**Figure 9:**
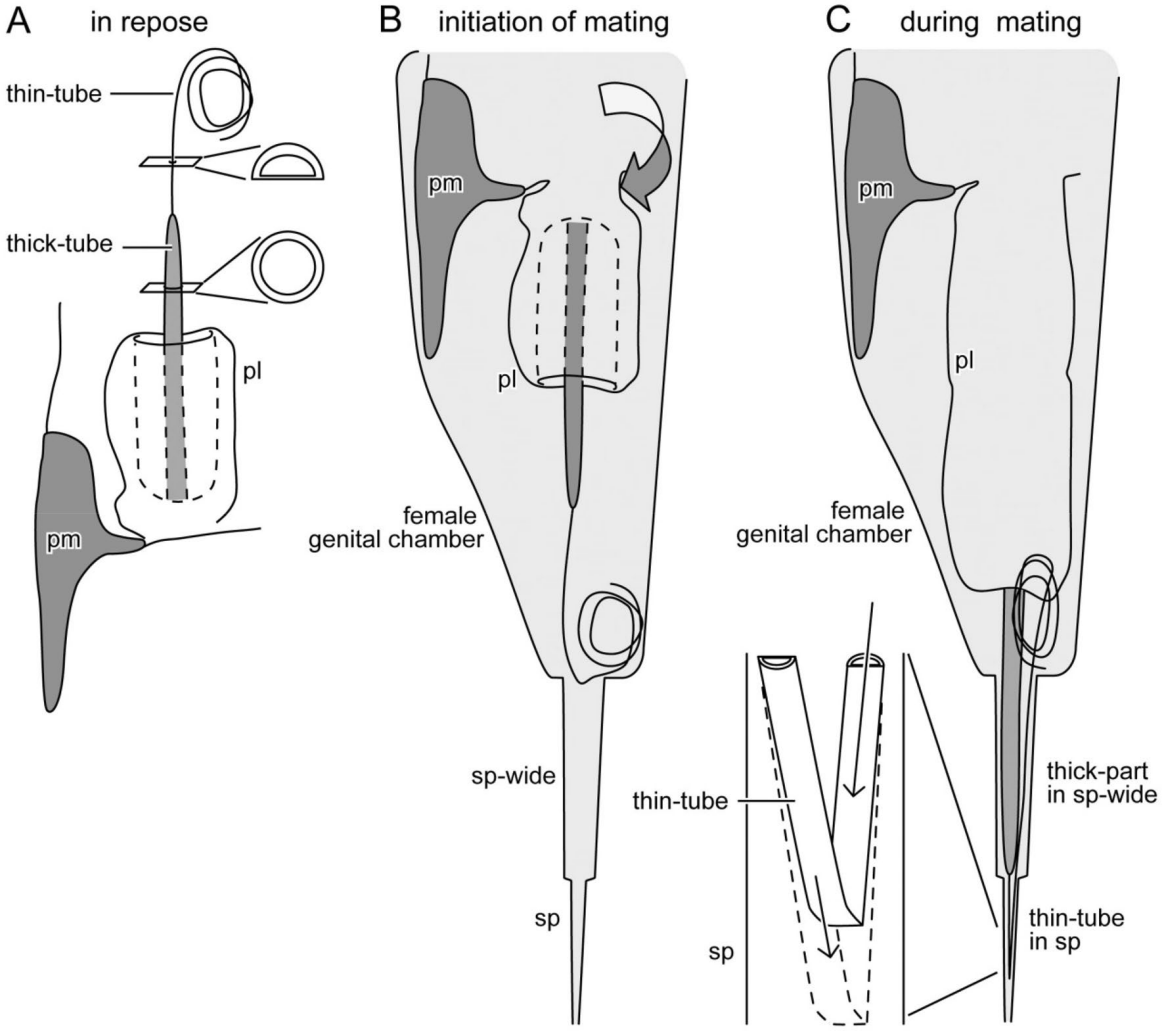
Schemes of the male genitalia of *Echinosoma horridum* and the hypothetical penetration mechanism of the virga. The left half is drawn. (**A**) The virga and the posterior half of the penis lobe is invaginated in its anterior half, and the entire penis lobe orients cranially in repose. At the initiation of mating, the penis lobe is unfolded and orients caudally in the female genital chamber (**B**), and then the invaginated part of the penis lobe and the virga are evaginated (**C**). At the same time, the thin-tube of the virga is folded and inserted into the spermatheca, and due to ejaculate pressure generated by the capillary action, the fold moves deeper and deeper into the spermatheca (**C**). Abbreviations: pl, penis lobe; pm, paramere; sp, spermatheca; sp-wide, the proximal and wide region of the spermatheca.

It appears to be a challenge for males to insert and withdraw the extremely slender virga (aspect ratio, 1:2117) into the spermatheca since the high aspect ratio can easily cause buckling of the structure. Although it is known that indeed such long virgae in other earwigs penetrate the spermatheca^34,35^, the insertion and withdrawal mechanisms remain unclear. The geometry and bending stiffness reported here on *E. horridum* suggest the following penetration mechanism (Fig. 9). (1) While either of two penis lobes is unfolded from cranially to caudally during mating (Fig. 9A), (2) the thin-tube base of the virga is folded due to a limited space of the female genital chamber and the spermatheca (Fig. 9B), and the fold is inserted to the spermatheca (Fig. 9C). (3) At the same time, the thin-tube of the virga, which is plausibly filled with sperm due to capillary action^36^, and the fold move deeply in the spermatheca by the capillary force (Fig. 9C). The possible withdrawal mechanism of the virga is that the male pulls the entire virga out by moving apart from a female mate, while the penis lobe is moved cranially to store the whole virga back to the male genital chamber probably by a combination of the decrease of haemolymph pressure and protractor muscles. Analogies of these hypothetical insertion and withdrawal mechanisms are known in the fruit fly *Ceratitis capitata*^37^ and the rove beetle *Aleochara tristis*^38^. Still, our explanation for *E. horridum* is merely a hypothesis, and the reproductive biology of this species has to be investigated.

This hypothetical insertion and withdrawal mechanisms can explain why neither geometric measurements (Fig. 7A) nor calculated bending stiffnesses (Fig. 8E) of the virga of *E. horridum* shows gradients or heterogeneity of material distributions, unlike the short virga of *E. denticulatum*. With this penetration mechanism, the prerequisite is high pliability of the virga. The bending stiffness of the thin-tube of the virga is markedly lower than the virga of *E. denticulatum* (Fig. 8E,F), and due to the semi-circular cross-section, the thin-tube tends to bend in one direction (Fig. 9A,C). Because of this feature, we could not break even the dried thin-tubes, which were just bent, unlike our other specimens.

Another noteworthy point we found is that the bending stiffness of the thin-tube of the virga is much lower than that of the spermatheca of *E. horridum*, but it is reverse in *E. denticulatum*. Filippov et al.^11^ numerically demonstrated that a stiff intromittent organ of males deforms a less stiff spermatheca during penetration and this penile behaviour leads to a slower penetration. By contrast, a stiff intromittent organ with a flexible apical region results in a comparatively quick penetration^11^, suggesting a smooth penetration. The combination of a flexible male and comparatively stiff female genitalia is also experimentally demonstrated in *Cassida rubiginosa*^12 ^(see Table 2) . Although to date, only two exemplary cases showed this relative stiffness combination, we consider this can be one of the universal principles of the penetration mechanics of slender and hyper-elongated intromittent organs.

In Table 2, we summarised findings and insights regarding the penetration mechanics of earwig and beetle genitalia. In this way, we may partially address the three questions posed in the introduction: (1) Are the functional principles limited to the beetle species? (2) Are they correlated to the genital length? (3) Do the functional principles depend on the mechanical properties of corresponding female structures? As discussed above, the functional principles found in the beetle were only partially applicable to the earwig species, and not all of them are associated with the genital length. The male genitalia are more flexible than the corresponding female genitalia in both species with the hyper-elongated penis. Interestingly, the bending stiffness gradient, i.e. the apical part is more flexible than the rest in the male, was found in *E. denticulatum* with the short virga and *C. rubiginosa* with a hyper-elongated penis. Irrespective of a huge difference in the length of the male genitalia of *E. denticulatum* and *C. rubiginosa*, it is conceivable that the bending stiffness gradient plays a vital role in the penetration mechanics due to their similar penetration mechanism.

The present and previous studies unveiled that a number of variable factors affect penetration mechanics, and we hope that further research will shed more light on the penetration mechanics. In addition to this, functions of female genital spines, setae and microtrichia, observed on the inner surface of the virga, are still entirely unclear. Although these do not seem to be directly related to the topic of the present study, these details could be important to understand the penetration process more comprehensively. The reproductive biology and morphology of earwigs have to be further examined. Earwigs would be a great candidate group to unveil the penetration mechanics from a comparative perspective due to their remarkable diversity of the virga and spermatheca morphology and function^25^. In Dermaptera, for example, it is known that the virga of *Euborellia plebeja* (Dohrn, 1863) (Anisolabididae) is used for sperm removal and that its tip is specialised to rake sperm^34^, whereas the virga tip of *Marava arachidis* (Yersin, 1860) (Spongiphoridae) is curved and slightly swollen in shape, and the virga may not be used for physical removal of rival sperm^35^. In future studies, they may be candidate species for comparison to *Echinosoma* species.

## Material and methods

### Specimens

*Echinosoma denticulatum* specimens were collected in forested areas of Bukit Jambul and Bukit Kukus, Penang Island, Malaysia and were kept in laboratory conditions at 26 ± 1 °C (12 h photoperiod)^21^. Samples of *E. horridum* were collected in forested areas in Perak and Selangor states, Malaysia and kept in similar condition as above. This field study was conducted in 2012 and 2013 with the approval of the Economic Planning Unit, Malaysia (Reference No. UPE: 40/200/19/2844). In total, we examined five males and five females of *E. denticulatum* and eight males and four females of *E. horridum*. We mainly used either anaesthetised animals or specimens preserved in a conventional freezer and also investigated some specimens preserved in 70% ethanol, as described below in detail. Before dissection, entire bodies of earwigs were photographed at different focus planes using a Leica M205A stereomicroscope equipped with a Leica DFC420 camera, and the photographs were stacked with the aid of the software LAS V3.8 (Leica Microscopy GmbH, Wetzlar, Germany). Based on the images, the body length, i.e. the length from the anterior end of the head to the distal tip of forceps (cerci), was measured using the software Fiji 1.53c^39^. All specimens were dissected in 0.1 M phosphate-buffered saline (PBS; pH = 7.4; Carl Roth GmbH & Co. KG, Karlsruhe, Germany) under an Olympus SZX12 stereomicroscope (Olympus Corporation, Tokyo, Japan).

### Genital length measurement

The genital length was measured based on micrographs using the software Fiji. The male virga lengths of *E. denticulatum* were measured based on CLSM images. By contrast, virgae of *E. horridum* and spermathecae of both species were complex, and it was impossible to measure the lengths based on CLSM images. Therefore, after CLSM imaging, five specimens of *E. horridum* virgae were uncurled and covered with cover glasses. Micrographs of the virgae were taken using a Zeiss Axioplan light microscope equipped with a Zeiss AxioCam MRc camera (Carl Zeiss Microscopy GmbH, Jena, Germany), and based on the micrographs, the lengths of the virgae were measured using the software Fiji. To measure the spermatheca lengths of both species, we submerged one sample of *E. denticulatum* and two spermathecae of *E. horridum* in 10% KOH to unfold folded and convoluted ducts.

### Geometrical characterisation

Some of the male and female samples used for the CLSM analyses were rinsed with distilled water and dehydrated with ascending ethanol series (10% to 99.5% ethanol, with each step 20 min, except for 99.5% ethanol, where the samples were kept for a night). Some samples were immersed in Hexamethyldisilazane (HMDS) and were kept at room temperature, ca. 20 °C, in a fume hood to evaporate the chemical. In contrast, other samples were dried at critical point using critical point driers (E3100, Quorum Technologies LTD, Kent, UK and Leica EM CPD 300, Leica Microscopy GmbH). Then, when possible, they were cut into either three (base, middle, apex) or four (thick-part, thin base, thin middle, thin apex in *E. horridum* males) regions using micro-scissors and blades. It was practically not possible to cut them into three or four segments with exactly same lengths, and segments closer to the base are defined as base and ones closer to the apex were defined as apex. Each segment was broken into small pieces and perpendicularly placed on SEM stubs to observe their cross-sections using a Hitachi S-4800 scanning electron microscope (Hitachi High-Tech Corp., Tokyo, Japan) at an accelerating voltage of 3 kV. All samples were sputter-coated with gold–palladium (ca. 10 nm in thickness) using a Leica EM SCD500 high vacuum sputter coater (Leica Microscopy GmbH) before observation. Based on resulting micrographs, we measured diameters and wall-thicknesses of male and female ducts using the software Fiji. The measurements were analysed using the software R.

The abovementioned SEM-based observations showed that cross-sections of the thin-part of the *E. horridum* virga are semi-circular. However, this shape could be an artefact resulting from the cutting of the virga using the micro-scissors. To confirm this observation, we also applied a Zeiss LSM 700 confocal laser scanning microscope (Carl Zeiss Microscopy GmbH, Jena, Germany) to other samples. For this purpose, two samples preserved in 70% ethanol were dissected in distilled water. The virgae were hydrated in distilled water for a while and submerged in glycerine (≥ 99.5%, free of water, Carl Roth GmbH & Co. KG) on glass slides. Between a cover glass and a glass slide, we glued transparent reinforcement rings to make a small gap and to avoid contacts between samples and the glasses following Michels and Buntzow^40^. A stable solid-state laser with a wavelength of either 488 nm or 555 nm was used for excitation. For the detection of selective emitted autofluorescence, a longpass emission filter, transmitting light with either wavelength ≥ 490 nm or ≥ 560 nm, was applied, respectively. An object lens with 20 × magnification (Zeiss Plan-Apochromat, air immersion, NA = 0.8) was used.

### Material distribution analyses

Virgae and spermathecae were dissected out in 0.1 M phosphate-buffered saline (PBS; pH = 7.4; Carl Roth GmbH & Co. KG) under an Olympus SZX12 stereomicroscope (Olympus Corporation) from male and female earwigs either anaesthetised with carbon dioxide or frozen. Then we submerged the virgae and spermathecae in glycerine on glass slides, and as mentioned above, placed a gap holder between the glasses, not to damage samples. To stabilise samples in glycerine, we kept the samples in a conventional refrigerator (4 ºC) at least for one night. Four stable solid-state lasers with wavelengths of 405 nm, 488 nm, 555 nm and 639 nm were used for excitation of autofluorescence. For detection of selective emitted autofluorescence, a bandpass emission filter, transmitting light with wavelengths 420–480 nm or longpass emission filters, transmitting light with wavelengths ≥ 490 nm, ≥ 560 nm or ≥ 640 nm were applied, respectively. An objective lens with either 10 × magnification (Zeiss Plan-Apochromat, numerical aperture 0.45) or 20 × magnification (Zeiss Plan-Apochromat, air immersion, NA = 0.8) was applied. Following Michels & Gorb^26^, we assigned blue, green, red (50% saturation) or red (50% saturation) to each image produced with the abovementioned four sets of lasers and filters. Then the resulting images were overlaid with maximal intensity projection using the software Zeiss Efficient Navigation (ZEN) (Carl Zeiss MicroImaging GmbH). We described the results of material stiffnesses following the colour code established by Michels & Gorb^26^ as mentioned above.

### Estimation of bending stiffness

We first estimated Young's modulus of male and female elongated genitalia based on their CLSM images using the programme developed by Eshghi et al.^28^. The programme establishes a quantitative link between the autofluorescence properties of the constituent components of insect cuticle and their Young's modulus. Briefly, the programme detects the primary colour that has the maximum intensity in each pixel in the CLSM image. The value of the intensity is multiplied by the corresponding Young's modulus of each primary (6.8 GPa, 1.5 GPa and 1.95 MPa for the red, green and blue primaries, respectively)^28^. The validity of the assigned Young's moduli has been tested by comparing experimentally measured Young's moduli of adhesive feet setae of a ladybird beetle^41^ to the estimated Young's moduli obtained by the programme^28^.

To estimate the Young's moduli in the male, using the programme, we first drew 30 uniformly distributed lines, each of which is composed of 20 points. And every 20 points were distributed only along the male genitalia. The Young's moduli were extracted for every pixel, where the points were located. The obtained Young's moduli were averaged once for each line, and then the data that were situated in each measurement location, i.e., base, middle and apex, were separately pooled. For the spermatheca, we extracted results of arbitrary 20–25 points on spermathecal walls from each specimen. Since soft tissues surround the spermathecal walls, the standard method applied to the male samples was not suitable for the female. For a quantitative comparison of the Young's moduli between the sexes, we used only CLSM images, in which male and female genitalia were scanned together.

The second moment of area for bending in the direction perpendicular to the longitudinal axis was calculated using the BoneJ plugin^27^ for Fiji. For this purpose, cross-section images of male and female genitalia, obtained with CLSM and SEM, were used. The cross-sections were traced manually with the software Illustrator CS5 (Adobe Systems, California, U.S.A.) to increase image contrast, and the resulting images were imported to Fiji equipped with the plugin BoneJ. Taken into account that cross-sections often resembled an annulus except for the thin-part of the virga of *E. horridum*, to ensure the accuracy of the results obtained by BoneJ, we calculated the second moment of area using the following equation:

$${\text{I }} = { }\frac{\pi }{4}\left( {r_{2}^{4} - r_{1}^{4} } \right)$$

where I is the second moment of area, $r_{2}$ is an outer radius and $r_{1}$ is an inner radius.

The Young's modulus of each measurement location in combination with their corresponding second moment of area was used to calculate the bending stiffness of that location.

### Statistics

All statistical analyses of the data were conducted using the 'R' software (ver. 4.0.2)^42^. Due to difficulty in sample preparation, we obtained measurements as many as we can from every individual. However, to avoid pseudoreplication, we calculated means for each individual, except for the data on the second moment of area where we pooled all measurements. With the data, we tested increasing or decreasing trends of measurements along the male and female genitalia using a Kendall's rank correlation coefficient. To compare data between the sexes, we used a Mood's median test when both the assumptions of normality (tested by a Shapiro–Wilk test) and homogeneity of variance (tested by a F-test) were rejected. When only the latter assumption was rejected, we compared the data using a Wilcoxon rank-sum test.

## Supplementary Information

Supplementary Information.

## Data Availability

Supporting data are available as supporting information.
