# Supplementary material for: Penetration mechanics of elongated female and male genitalia of earwigs
Source: Sci Rep. 2021 Apr 12;11:7920. doi: 10.1038/s41598-021-86864-1 (PMC8041768; doi:10.1038/s41598-021-86864-1)
Supplement: Supplementary file 1 — Supplementary Information. [file 41598_2021_86864_MOESM1_ESM.pdf]

Supporting information (Tables 1-6, Figs. 1-6)

Penetration mechanics of elongated female and male genitalia of earwigs

Yoko Matsumura[1]*, Yoshitaka Kamimura[2], Chow-Yang Lee[3,4], Stanislav N Gorb[1,⁋], Hamed Rajabi[1,⁋]

⁋ Stanislav N Gorb and Hamed Rajabi share last authorship.

[1] *Department of Functional Morphology and Biomechanics, Zoological Institute, Kiel University, Am Botanischen Garten 1–9, D-24118 Kiel, Germany*

[2] *Department of Biology, Keio University, 4-1-1 Hiyoshi, Yokohama 223-8521, Japan*

[3] *Urban Entomology Laboratory, Vector Control Research Unit, School of Biological Sciences, Universiti Sains Malaysia, Minden 11800, Penang, Malaysia*

[4] *Present address: Department of Entomology, University of California, Riverside, California, USA*

* Corresponding author: yoko.matumura.hamupeni@gmail.com

Table 1. Outer diameters of male virga (mean ± sd µm [sample numbers]) of *Echinosoma denticulatum*. The letters correspond to locations, whose diameters were measured and shown in schemes of the virga and spermatheca (Fig. 7F).

	E	F	G
Echinosoma denticulatum	40.83 ± 3.62 (5)	32.23 ± 3.92 (12)	26.91 ± 2.36 (11)

Table 2. Outer diameters of male virga (mean ± sd µm [sample numbers]) of *Echinosoma horridum*. The letters correspond to locations, whose diameters were measured and shown in schemes of the virga and spermatheca (Fig. 7E).

	H	I	J	K	I+J	J+K
Echinosoma horridum	43.20 ± 7.87 (11)	6.01 ± 1.12 (8)	NaN	NaN	6.38 ± 0.51 (2)	4.02 ± 0.66 (2)

Table 3. Wall thicknesses of male virga (mean ± sd µm [sample numbers]) of *Echinosoma denticulatum*. The letters correspond to locations, whose wall thicknesses were measured and shown in schemes of the virga and spermatheca (Fig. 7F).

	E	F	G
Echinosoma denticulatum	5.64 ± 1.34 (29)	6.08 ± 1.30 (31)	6.15 ± 0.67 (21)

Table 4. Wall thicknesses of Outer diameters of male virga (mean ± sd µm [sample numbers]) of *Echinosoma horridum*. The letters correspond to locations, whose wall thicknesses were measured and shown in schemes of the virga and spermatheca (Fig. 7E).

	H	I	J	K	I+J	J+K
Thickness 1	6.28 ± 1.30 (61)	0.62 ± 0.23 (17)	0.55 ± NA (1)	0.51 ± NA (1)	0.71 ± 0.088 (3)	0.48 ± 0.12 (5)
Thickness 2	NaN	0.28 ± 0.12 (13)	0.28 ± 0.062 (2)	0.15 ± NA (1)	0.26 ± 0.031 (3)	0.25 ± 0.080 (5)

Table 5. Outer diameters of females spermathecal ducts (mean ± sd µm [sample numbers]). The letters correspond to locations, whose diameters were measured and shown in schemes of the virga and spermatheca (Fig. 7E,F).

	A	B	C	D	B+C+D
Echinosoma denticulatum	40.35 ± NA (1)	15.76 ± 0.90 (5)	15.32 ± 1.10 (8)	15.12 ± 1.70 (4)	16.20 ± 1.68 (70)
Echinosoma horridum	NA	11.46 ± 1.11 (23)	12.05 ± 1.47 (16)	14.46 ± 2.62 (9)	12.66 ± 1.72 (158)

Table 6. Wall thicknesses of female spermathecal ducts (mean ± sd µm [sample numbers]). The letters correspond to locations, whose wall thicknesses were measured and shown in schemes of the virga and spermatheca (Fig. 7E,F).

	A	B	C	D	B+C+D
Echinosoma denticulatum	4.20 ± 0.44 (2)	1.45 ± 0.38 (7)	1.43 ± 0.20 (7)	1.20 ± 0.57 (5)	1.71 ± 1.49 (122)
Echinosoma horridum	NA	0.44 ± 0.094 (45)	1.25 ± 0.39 (19)	1.91 ± 0.66 (13)	1.14 ± 0.83 (317)

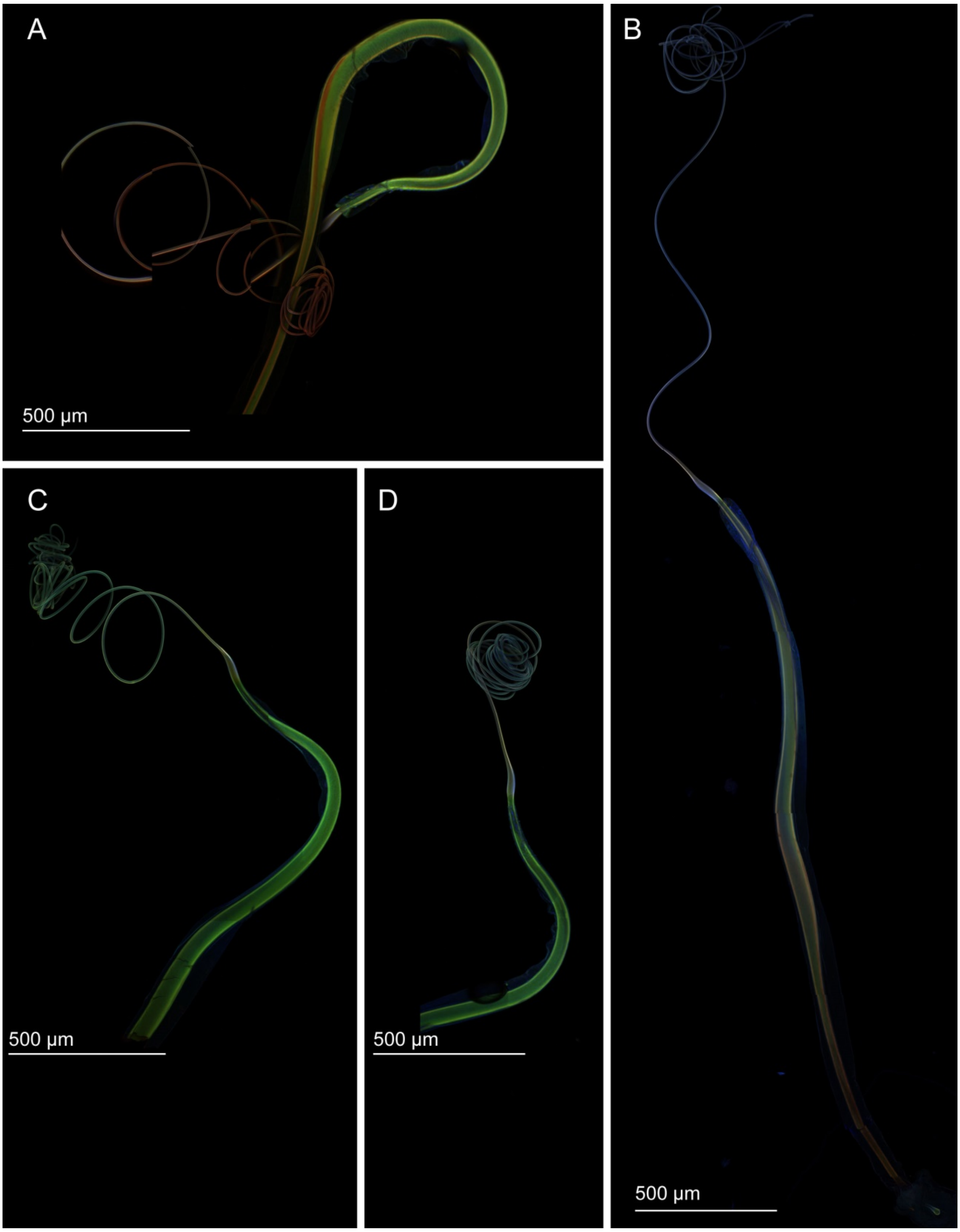

Supporting information Fig. 1. Confocal laser scanning micrographs of the virga of *Echinosoma horridum*, showing intra-specific variations of material gradients.

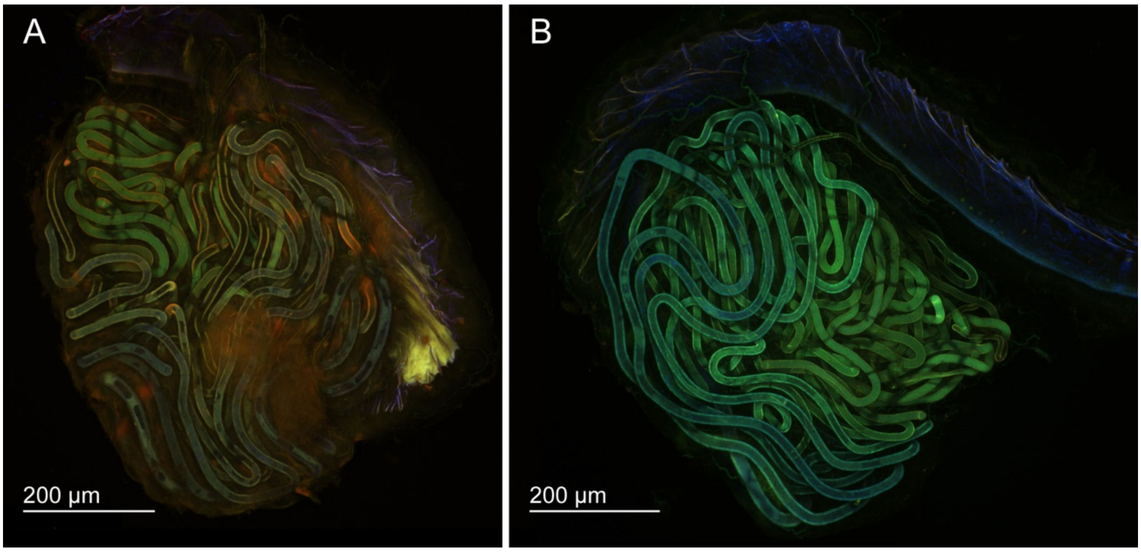

Supporting information Fig. 2. Confocal laser scanning micrographs of the spermatheca of *Echinosoma horridum*, showing intra-specific variations of material gradients.

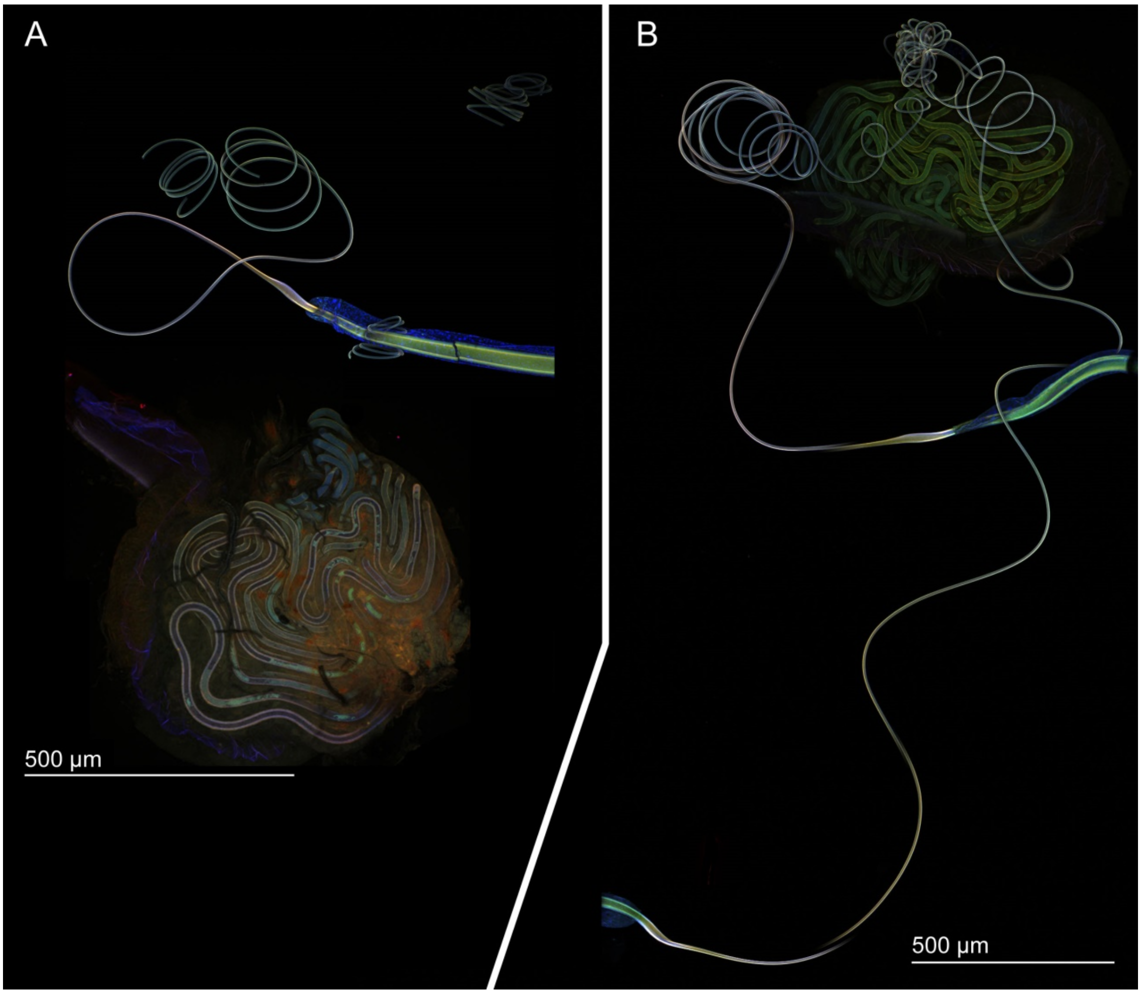

Supporting information Fig. 3. Confocal laser scanning micrographs of the virga and spermatheca of *Echinosoma horridum*, showing intra-specific variations of material gradients.

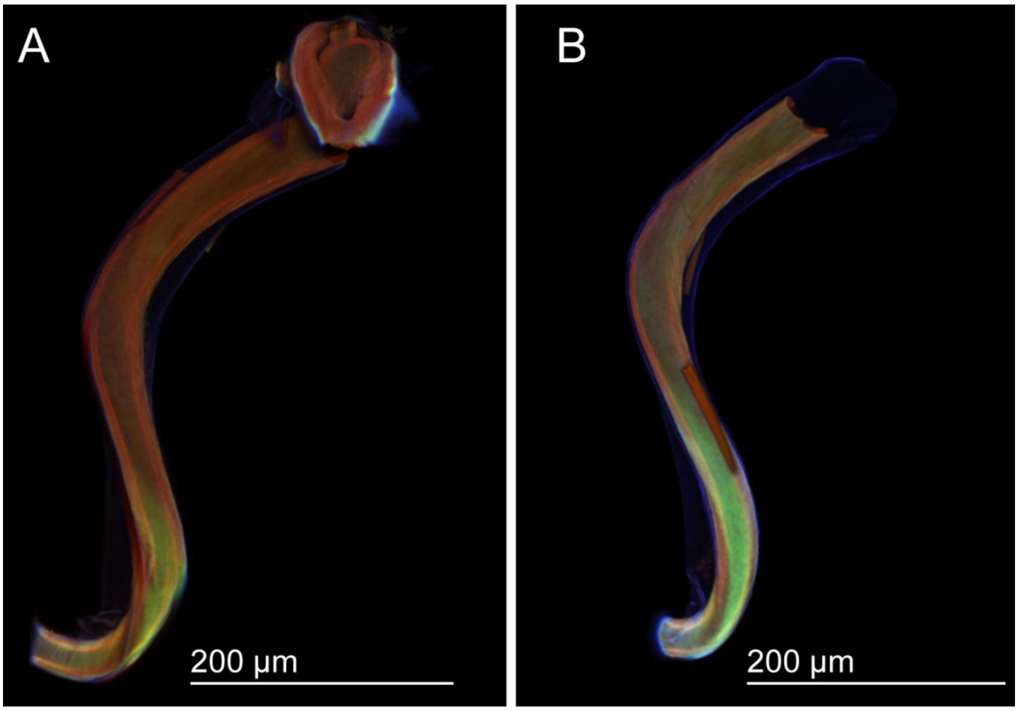

Supporting information Fig. 4. Confocal laser scanning micrographs of the virga of *Echinosoma denticulatum*, showing intra-specific variations of material gradients.

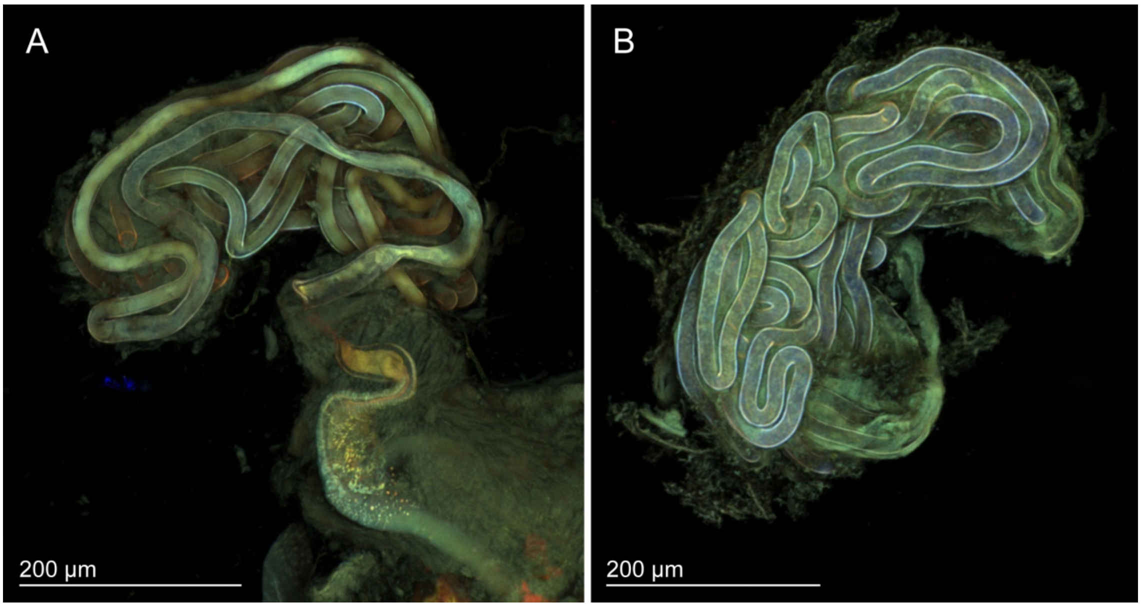

Supporting information Fig. 5. Confocal laser scanning micrographs of the spermatheca of *Echinosoma denticulatum*, showing intra-specific variations of material gradients.

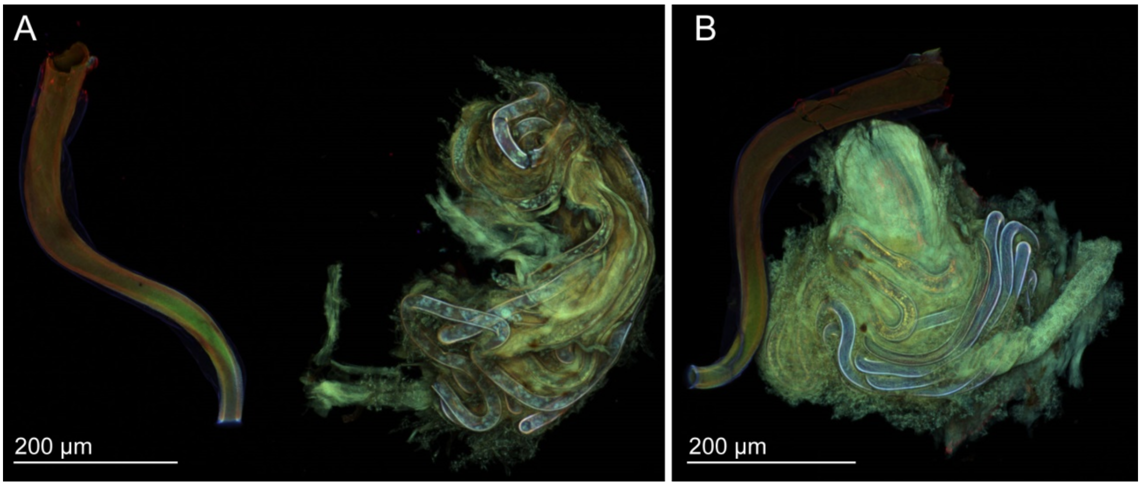

Supporting information Fig. 6. Confocal laser scanning micrographs of the virga and spermatheca of *Echinosoma denticulatum*, showing intra-specific variations of material gradients.
